# Remedies for Diarrhœa

**Published:** 1866-10

**Authors:** 


*Remedies for Diarrhœa.*—We proceed, as we promised, to give a comparative view of the remedies for bowel disorder. In so doing we go on the positive, not on the negative plan. That is to say, if practitioners of experience report that any given class of remedies proves efficacious in their hands, we accept the fact; and if other practitioners report good results from an opposite class of remedies, we accept that fact also. And we take liberty to use the solution of this seeming paradox which common sense and experience furnish us with. That is to say, we assume what we know to be true, that the cases in which opposite remedies are respectively used belong to opposite classes of cases, or else that divers remedies may attain the same end in the same class of cases by opposite routes.

The remedies which we propose to consider—shortly and in the space of one essay—were enumerated in a late article, and we give them again, in alphabetical order.

Acids, Alkalis and Absorbents, Antiseptics, Astringents, Calomel, Meat, Opiates, Purgatives, Stimulants.

The questions we have in view are these: Supposing a man has to treat a crowd of patients affected with diarrhœa, what is the best general, shall we say routine, remedy? What, if used indiscriminately, will do good in the greatest number, and harm in the least number of cases? And on the other hand, supposing time and circumstances allow of a discriminate and individual application of remedies, what are the cases for which each remedy is best adapted?

We must also say, that we are discussing remedies for diarrhœa, and possibly for cholerine (this one word *cholerine* is better than *choleraic diarrhœa*), not for cholera, *i. e.*, the stage of collapse; further that the absolute repose and warmth of bed is a means of safety which it is difficult to over-estimate; and besides, as our correspondent said, in his account of a visit to Amiens, it seems possible that a patient who is *cured* of diarrhœa shall yet die of cholera.

1. The acids are the more worthy of trial by those who have never tried them, inasmuch as they seem at first to run counter to all our English opinions and traditions. Yet lemon juice and salt is the old and popular remedy in Italy and the East for diarrhœa. Traces also of treatment by acids are found in the writings of some practical physicians of the last century. But, so far as we know, the person who first brought them into vogue in England was an obscure practitioner named Coleby, in Bermondsey, who gained a reputation and a fortune by his use of dilute nitric acid in large doses in the premonitory diarrhœa during the cholera epidemic of 1832. Since that time the sulphuric acid has come into vogue, and its use has been advocated by many practitioners, as Mr. Boddington and Mr. Tucker, and it has now become so popular that it cannot be considered as the speciality of any one. Any acid appears to answer—sulphuric, nitric, phosphoric, hydrochloric, tartaric or citric. Mr. Tucker pointed out, in 1854, the value of *cider*, with its malic, acetic, and tannic acids; but the sulphuric seems really the best and most convenient. The right way of using an acid for diarrhœa is not to give it in any measured dose, but to cause it to be mixed with water, sweetened and flavored with ginger—say half a drachm of dilute sulphuric acid, two drachms of syrup, and a half a tumbler of cold water. The patient may dilute it and sweeten it to the precise point he likes, and be told to drink it freely in as great a quantity as he likes; but few care for more than three drachms in the twenty-four hours. The effect of the remedy seems direct and positive. It stops diarrhœa, quells pain, puts an end to the generation of flatulence, and leaves the patient well. Most positively there is nothing purgative in its action. It is suited for atonic diarrhœa, with clean tongue, in every stage. Where diarrhœa has been going on, leaving the belly puffy with griping pains and flatulence, the acid acts like a charm. Again, in the cases of diarrhœa which effect over-fed people—with foul tongue, yellow eye, and loaded liver (as we assume)—the acid is a good remedy by itself, but better if employed as an adjunct to a dose of calomel. Moreover, as a prophylactic, as Dr. MacCormac has shown, it is of great value. The minute that a man says he can't eat through the heat, and lives upon beer, he should be advised to take some mineral acid in bitter infusion. It will not be taken for granted that there are no cases in which it fails. In some it fails, apparently without reason; and in cases where there is a red, irritable tongue, it should not be tried. But there is one almost certain indication—that if the patient likes it, it is almost sure to cure him; if he dislikes it, it will almost surely do him no good. The acids, moreover, are adapted for all ages and conditions, from the infant at the breast with chronic, or atonic, or serous diarrhœa, to the veteran in his second childhood. The Elixir of Vitriol, or aromatic sulphuric acid, is nicer, and well adapted for private practice; but the common pure acid is quite good enough for most cases.

2. There are the alkalis and alkaline earths, as chalk, to which, we suppose, bismuth must be added. There is no doubt that these remedies have a certain force, though not much in severe cases. They deodorize, or neutralize, or alter the quality of the intestinal mucous membrane, and (if employed alone) are suitable for infants and persons with red tongues and irritable, possibly ulcerated, bowels. The dose of chalk or bismuth should be very much larger than is commonly given, say thirty grains every hour—quite enough to produce a decided chemical change in the contents of the alimentary canal, and to be detected in the fæces.

3. Antiseptics—*e. g.*, creosote, in doses of one drop in pills, is good in many an atonic diarrhœa. Its *modus operandi* seems clear.

4. Astringents. Under this head we have the vegetable astringents, as gallic acid, catechu, tormentil, etc., adapted for atonic diarrhœa with clean tongue. But it must be observed that these remedies are seldom given without opium, and that they do not appear superior to sulphuric acid—certainly not so good for popular use. Of mineral astringents, the acetate of lead, in two or three large doses, is most in vogue, especially with Indian practitioners.

5. Calomel is the English remedy *par excellence* for all cases attended with foul loaded tongue, yellow eye, thirst, headache, etc. We believe that it really has the power of checking pale serous stools, and of producing thick offensive ones, and in the right cases is without doubt the most powerful and certain remedy we have. In the opposite class it is equally mischievous.

6. Raw meat pounded into a pulp is a remedy which we first saw used by Professor Trousseau, at the Hopital des Enfants Malades, in Paris, nearly twenty years ago. It is especially adapted for young infants, who take it greedily; it appears to be digested without effort, and, by giving healthy material to the blood, cuts off those causes of diarrhœa which consist in an impoverished state of blood. Its specialty is as a remedy for infants and children with whom nothing else agrees or is digested. But it is also the food *par excellence* for any case whatever at any age in which food can be taken. Raw mutton is the thing—deprived of skin and fat, and pounded in a pestle and mortar till it is a mere paste. This may be given with or without a very little sugar to infants, in a dose of a teaspoonful at a time; adults may take a larger quantity diffused through cold beef-tea. We have established the fact to our own satisfaction that meat *raw* differs from meat cooked in being more digestible, although meat cooked, if pounded to a powder and used to thicken broth, is a most useful food for adults, so soon as food is permissible.

7. *Opium.*—That this blessed and priceless antidote to human woe should be useful in diarrhœa, is no more than might be expected from its power of allaying irritation and pain and local afflux of blood. And the largest experience shows that with an ill-fed, ill conditioned, below par population small doses of opium act like a charm in a large percentage of cases of atonic diarrhœa with a clean tongue. Its action and use in such cases are consistent with the whole science and practice of Medicine and Surgery. Let us suppose that the *bowels are loaded*, and that there are diarrhœa, pain and spasm. The bowels are clearly unequal to the task of efficiently and vigorously discharging their contents; and in giving opium, the practitioner does what the surgeon does when a patient with an irritated bladder cannot pass his urine, and what the accoucheur does when a woman is plagued with inefficient uterine action. Opium rests a weary colon, and allows it time to gather strength to discharge its duties with vigor. There are two classical works on surgery—Vincent's "Surgical Observations," and Hilton's "Lectures on the Principle of Rest;" both concur in advising the use of measures to allay fretfulness of the whole body and of parts, and to give time. Any one who denies the value of opium in atonic painful diarrhœa, must be put out of the category of arguable persons. Equally ought that man be sent to coventry who affirms that it is a universal remedy. Opium used indiscriminately in cases in which acids or calomel or purgatives ought to be used—that is, in cases where the tongue is coated and foul and there are evidences of loaded liver or *embarras gastrique*—may make the patient ill for weeks. We believe that we have seen fatal results follow the summary suppression, by opium, of a salutary diarrhœa, in over-fed, gormandizing, red-faced, tight-bellied personages, especially women.

8. *Purgatives.*—When diarrhœa occurs to persons who have previously suffered from constipation, in whom there are evidences of loaded bowels—fœtid, lumpy, quasi-dysenteric stools—the belly showing by palpitation and percussion signs of accumulation in the colon, and the mouth and tongue nauseous, then the practitioner need seldom refuse to venture on a dose of rhubarb and polychrest salt or castor oil, preceded or not by a dose of calomel. When the motions are inodorous, and the belly is empty, the use of purgatives is simply cruel.

9. Stimulants, such as ether, essence of camphor, the essential oils, as peppermint, cayenne pepper, and more especially the almost too familiar prescription of a hot and strong glass of brandy and water, are of well known efficacy in almost all cases of diarrhœa, except those attended with marked *embarras gastrique.*

If we may sum up our own impressions of *single* remedies, we give the first place to acids, as being more serviceable and less noxious than any other in the greatest number of cases; but for cases attended with well marked symptoms of pain, they are inferior to opium, in those with *embarras gastrique* to calomel, and in those of fæcal accumulation to calomel, or rhubarb with polychrest salt. Still they are the best single or universal remedy.

Whilst the science of therapeutics demands the employment of remedies singly, the necessities of practice compel combination, in order to secure different or even opposite effects, and so to hasten the patient's cure. The acids may be used as auxiliaries to any other remedy if the patient relish them. Opium may be combined with stimulants in atonic painful diarrhœa, and the popularity of the compound called chlorodyne shows it to be a useful model for imitation now, just as itself is an imitation of the ancient cordials and theriacas. Opium, in fact, which soothes pain, may be combined with eliminative remedies, so as to add the *tuto* and *jucunde* to the *cito.* Of such combinations the time-honored one of calomel two grains, opium half grain, is one which scarcely needs our praise. For a severe case it probably is the best thing that can be prescribed. It may also be combined with acids, or with alkalis, absorbents, astringents and stimulants, as in the well known compound chalk powder with opium.

The other day, happening to talk with a lady of rank on the cholera, we were asked, "Do you belong to the brandy faction, or the calomel faction?" We hope that our foregoing remarks have shown that there need be no factions at all, and that each remedy is good in its place, and that that place is pretty well known to practical men who are not committed to any faction. —*Lond. Med. Times and Gazette*, July 28, 1866.

---

*Cholera.*—The Edinburgh (Scotland) medical officer of health, Dr. Littlejohn, with a staff of ten assistants, lime-washers, etc., makes a house to house visitation in the more densely populated parts of the city, compelling the dwellers in unclean places to turn out and get renovated. The Doctor has no small job on hand in the purlieus of what was once the shade of aristocracy.

We extract from the *Lancet* the following summary of a paper by C. B. Fox, M. D., on the "Sympathy between the Auditory Canal and the Larynx:"

1. The sympathy between the ear and the larynx as well as the stomach, has been long known, although the majority of recent writers seem to have overlooked it.

2. This sympathy is not manifested in every individual, but in about 17 per cent., and seems to depend on a state of hyperæsthesia of the nerve which supplies the auditory canal.

3. The nerve of the ear concerned in the production of this phenomenon cannot be a branch of the vagus, as Romberg and Toynbee have affirmed, but is, in all probability, a branch of the fifth cranial nerve.

4. This sympathy is an example of a reflected or sympathetic sensation, in which the connexion between the nerves concerned takes place in the nervous centre.

5. Cases occasionally occur where a cough is solely dependent on the existence of some source of irritation in the auditory canal.

6. The explanation of the sympathy between the ear and the larynx enable us to understand the mode in which pain of the ear becomes occasionally a symptom of a thoracic aneurism.

One of my chief objects in bringing before the notice of my professional brethren this sympathetic connexion is to introduce to them what may be called an *ear-cough*, and to strongly advise them to examine the auditory canals in all cases of obstinate cough, where none of the more frequent causes of this symptom can be discovered.

